# Novel Approaches in Food Preservation and Their Impact on the Food System

**DOI:** 10.3390/foods12214046

**Published:** 2023-11-06

**Authors:** Maria Tufariello, Marco Iammarino, Fabio Licciardello, Annalisa Mentana

**Affiliations:** 1 CNR—Institute of Sciences of Food Production (ISPA), via Prov. Lecce-Monteroni, 73100 Lecce, Italy; maria.tufariello@ispa.cnr.it; 2 Istituto Zooprofilattico Sperimentale della Puglia e della Basilicata, via Manfredonia 20, 71121 Foggia, Italy; marco.iammarino@izspb.it; 3 Department of Life Sciences, University of Modena and Reggio Emilia, 42122 Reggio Emilia, Italy; fabio.licciardello@unimore.it; 4 Interdepartmental Research Centre for the Improvement of Agri-Food Biological Resources (BIOGEST-SITEIA), University of Modena and Reggio Emilia, 42122 Reggio Emilia, Italy

The increase in consumer demand for safe, convenient, and fresh food with an extended shelf life is accompanied by an interest in the environmental impacts caused by the food industry. For example, the electricity used in refrigeration equipment is still mainly generated via the combustion of fossil resources, which significantly contributes to ozone depletion, pollutants, greenhouse gas emissions, and, overall, climate change. Also, the industry’s substantive use of non-biodegradable petroleum-based plastic packaging materials, necessary to protect food products, is increasingly causing the accumulation of macro- and micro-plastics in our environment. As a result, this crisis calls upon institutions and the global society for urgent solutions. Furthermore, heat-based processes, including pasteurization, sterilization, drying, and evaporation, are still the most common industrial practices that ensure the microbiological and physicochemical stability of foods: heat losses, reduced heat transfer efficiency, and thermal damage represent some of the drawbacks of these conventional technologies, raising the need for implementing novel technologies at the industrial level. Finally, replacing harmful food additives with novel technologies of food stabilization has gained significance in food research in response to consumers’ concerns about added chemicals in food [1].

We are pleased to present this Special Issue belonging to the “Food Packaging and Preservation” section, which covers six papers highlighting important research initiatives in the field of new approaches in food preservation as alternative and sustainable innovations in the food system. As guest editors, we present a brief overview of these contributions.

Cejudo et al. [2] presented a study focusing on the influence of supercritical CO_2_ treatment on stabilizing and storing white grape musts, addressing the influence of time, pressure, and CO_2_ percentage on must characteristics. The results showed that the CO_2_ level was the variable that most influenced the process and that the employed treatment did not significantly affect some key parameters, such as pH, acidity, and color intensity. In addition, this technique proved very effective at reducing aerobic mesophilic microorganisms and residual polyphenol oxidase activities, being lower than those obtained via SO_2_ addition (60 and 160 mg/L). As a result, the optimal conditions were 100 bar and 10% CO_2_ for 10 min of treatment.

Tomaiuolo et al. [3] described an approach based on UHPLC-Q-Orbitrap-MS and multivariate statistics to define the lipid fingerprint of Camembert cheese and to explore its correlated variation concerning X-ray irradiation. This treatment belongs to non-thermal technologies and represents a valid clean and safe alternative to preserve the hygienic quality of food and to extend the shelf-life of several foodstuffs, including dairy products. The results provided the lipid profile of Camembert cheese, identifying 479 lipids classified in 16 different lipid subclasses. Moreover, this study verified that the employed X-ray dose (3 kGy) did not lead to the formation of specific oxidized lipids or new lipid molecules related to this treatment. On the other hand, chemometric modeling, specifically partial least squares discriminant analysis (PLS-DA) and linear discriminant analysis (LDA), enabled the discrimination of irradiated versus non-irradiated samples and the selection of 42 lipids as potential treatment markers.

Two papers reported the results of novel processing technologies to extend the shelf life of meat and fish [4] and sour cherries [5], respectively.

Damdam and coworkers [4] evaluated the impact of UV-C irradiation and vacuum sealing in preventing the microbiological spoilage of beef, chicken, and salmon fillets. A constant UV-C irradiation dose of 360 J/m^2^ coupled with a reduced pressure of 40 kPa was used. The results demonstrated a statistically significant difference (*p* > 0.05) in aerobic bacteria counts between storage conditions and days in all samples, which is a primary indicator of microbial spoilage. However, there was no significant difference in the count of *Pseudomonas* spp. The combination of UV-C irradiation and vacuum sealing effectively inhibits microbial growth and extends the shelf-life of beef, chicken, and salmon fillets by 66.6%.

The influence of high-pressure treatment (HPP) on microbial stabilization and chemical–nutritional properties of pitted sour cherries was evaluated by Tenuta et al. [5]. The tested conditions (600 MPa for 3 min at 4 °C) were effective at minimizing the initial microbial load, which was maintained at negligible levels for 5 months of refrigerated storage. Regarding the nutritional aspect, the color and total phytochemical content of the black cherries remained at levels comparable to those of the fresh product for 3 months of refrigerated storage. Antioxidant activity, measured using ABTS and DPPH assays, was not affected by HPP treatment but was slightly reduced during refrigerated storage. Therefore, the study highlights the key role of HPP in prolonging the shelf life of sour cherries while safeguarding their quality.

Meat spoilage due to natural enzymatic processes and bacteria was also studied by Olivas-Méndez et al. [6], who evaluated the effectiveness of natural biocides found in essential oils and chili pepper oleoresins. Rosemary and garlic essential oils and pepper oleoresin, alone or in combination, as preservatives for beef burgers were evaluated. The results showed that the chosen ingredients, alone or in combination, improve microbiological quality and inhibit the lipid oxidation of beef burgers.

Finally, this Special Issue includes a paper focusing on the impact of three different combinations of sous vide (SV) on fatty acid composition, anserine and carnosine content, and sensory quality of pikeperch fillets (*Sander lucioperca*) [7]. The results showed that SV75 at 75 °C for 20 min and SV90 at 90 °C for 10 min are recommended for preparing pikeperch fillets, accounting for nutritional and safety aspects.

We sincerely hope this Special Issue will inspire and guide further research regarding changes in food preservation solutions. New political and scientific challenges must now be oriented towards technological change for sustainability.

## Data Availability

The data that support the findings of this paper are the articles published in this aforementioned Special Issue. These data were derived from the following available resources: https://www.mdpi.com/journal/foods/special_issues/approaches_food_preservation (27 January 2021).
